# A Vehicle Steering Recognition System Based on Low-Cost Smartphone Sensors

**DOI:** 10.3390/s17030633

**Published:** 2017-03-20

**Authors:** Xinhua Liu, Huafeng Mei, Huachang Lu, Hailan Kuang, Xiaolin Ma

**Affiliations:** School of Information Engineering, Wuhan University of Technology, Wuhan 430070, China; pelsor.whut@gmail.com (H.M.); huachang_cc@whut.edu.cn (H.L.); kuanghailan@whut.edu.cn (H.K.); maxiaolin0615@whut.edu.cn (X.M.)

**Keywords:** vehicle steering recognition, sensors, smartphone, context-aware system

## Abstract

Recognizing how a vehicle is steered and then alerting drivers in real time is of utmost importance to the vehicle and driver’s safety, since fatal accidents are often caused by dangerous vehicle maneuvers, such as rapid turns, fast lane-changes, etc. Existing solutions using video or in-vehicle sensors have been employed to identify dangerous vehicle maneuvers, but these methods are subject to the effects of the environmental elements or the hardware is very costly. In the mobile computing era, smartphones have become key tools to develop innovative mobile context-aware systems. In this paper, we present a recognition system for dangerous vehicle steering based on the low-cost sensors found in a smartphone: i.e., the gyroscope and the accelerometer. To identify vehicle steering maneuvers, we focus on the vehicle’s angular velocity, which is characterized by gyroscope data from a smartphone mounted in the vehicle. Three steering maneuvers including turns, lane-changes and U-turns are defined, and a vehicle angular velocity matching algorithm based on Fast Dynamic Time Warping (FastDTW) is adopted to recognize the vehicle steering. The results of extensive experiments show that the average accuracy rate of the presented recognition reaches 95%, which implies that the proposed smartphone-based method is suitable for recognizing dangerous vehicle steering maneuvers.

## 1. Introduction

The factors causing traffic accidents are many, among which human factors are dominant. In a study made by the American Automobile Association (AAA) Foundation for Traffic Safety, as many as 56% of deadly crashes involved one or more vehicle maneuvers typically associated with aggressive driving [1]. These actions include erratic lane-changes, making improper turns and excessive speeding. These maneuvers seriously endanger both the driver and public transport. Therefore, an important means to reduce the probability of traffic accidents is by reminding the drivers that dangerous vehicle maneuvers maybe exist around the road, thereby enhancing the driver’s safety awareness.

Steering-assistance systems, such as lane-departure warning or lane-keeping assistance, are typical examples. They all exploit the advanced built-in sensors (e.g., cameras, radars, and infrared sensors) to detect the lane for driving assistance [2,3,4,5]. However, these methods are subject to the influence of environmental elements or the high costs of hardware so these safety solutions cannot be applied to a wide range of type/year car models.

In the mobile computing era, smartphones have become instrumental tools to develop innovative mobile context-aware systems because of their numerous sensors such as GPS, accelerometers, gyroscopes. This makes them suitable enablers to capture a wide range of contextual features, like weather [6] and traffic [7] conditions, or user behaviors [8]. Consequently, they are key tools to develop ubiquitous mobile solutions [9,10].

In this paper, we make use of the inertial sensors existing on smartphones to take one step forward by developing a real-time accurate recognition system for dangerous vehicle steering maneuvers without the driver’s involvement. The basic idea is to use the phone’s gyroscope data to capture differences in the vehicle’s angular speed due to vehicle steering maneuvers, including turns, lane-changes and U-turns. To facilitate such a system design, several key challenges must be addressed:

(1) Smartphone posture alignment: we need to understand the smartphone’s posture before proceeding to the gyroscope data collection, otherwise the vehicle state will not be accurately derived;
(2) Steering time detection: to automate the initialization of a vehicle steering, it is critical to pinpoint the steering time at the starting point;
(3) Vehicle steering maneuver recognition: we need to recognize the steering maneuver of the situation that is based on the vehicle’s angular speed and the surrounding environment conditions.

To obtain the accurate but changeable information about the angular speed of the vehicle, we first correct the gyroscope data by rotating the matrix of the smartphone’s coordinate system so as to align the vehicle’s coordinate system. Then we use the endpoint detection algorithm based on parameters of the short-term energy spectrum to determine the occurrence of steering maneuver. Finally, we choose the matching algorithm of vehicle’s angular velocity based on Fast Dynamic Time Warping (FastDTW) to recognize the vehicle steering maneuver, unlike previous works [11,12,13], we also consider the condition of turn signal, the angle change in vehicle’s heading and the real-time weather data. Specifically, the following contributions are made in this work:

1. We propose a smartphone-based recognition system for vehicle steering maneuvers including turns, lane-changes and U-turns, and these maneuvers are characterized by gyroscope data from a smartphone mounted in the vehicle.
2. We design a new algorithm for the recognition of vehicle steering maneuvers. The algorithm can recognize different steering maneuvers by combining the vehicle’s angular velocity, the condition of turn signals, the heading angle change and the weather conditions, which improves the recognition accuracy.
3. We align the gyro’s coordinate system with the vehicle’s coordinate system by rotating the gyro’s coordinate system twice.
4. Extensive experiments are conducted and the experimental results demonstrate the feasibility and efficiency of our recognition system.

The remainder of this paper is organized as follows: Section 2 provides an overview of the state of the art of vehicle-maneuver recognition. The system overview and its related algorithms will be described in detail in Section 3. Then, Section 4 shows the final evaluation of the system. Finally, Section 5 puts forward the main conclusions of the work.

## 2. Related Work

Recognition technology for vehicle maneuvers has emerged in recent years. With the growing influence of intelligent transportation, this technology has developed rapidly for its own various availability in recent years, and has attracted many researchers’ attention. Existing recognition technology for vehicle maneuvers mainly includes three categories: video-based, vehicle sensor-based and smartphone sensor-based. 

### 2.1. Based on Video

Video-based recognition technology achieves the goal of identifying vehicle maneuvers through cameras which can capture images of the inside and outside environment of vehicles and the facial features of the drivers, through appropriate image segmentation processing technology. Jain from Cornell University installed a video camera and mobile computing devices around the rear-view mirror of a car [2]. The video camera can be used to capture traffic information and drivers’ facial characteristics during steering or lane-changes. Combined with vehicle GPS information, Hidden Markov Models (HMM) can successfully predict vehicle maneuvers. Video-based recognition technology is rather mature at present, displaying a relatively high recognition accuracy, but it is easily affected by the environmental conditions. If applied in a shadow covered, or damaged road, or in a backlit environment, its recognition rate will decline quickly, what’s more, it can’t be used for night driving. Due to the high environmental dependence of the video-based recognition technology, researchers have turned to look for other recognition strategies.

### 2.2. Based on Vehicle Sensors

Jiang’s CARLOG system is a framework between the built-in sensors of vehicles and a phone app [3,4]. Though collecting the data from vehicle sensors and using the Datalog language, the system outputs the form of predicates defining dangerous vehicle maneuvers to the computer, so as to identify vehicle maneuvers. In the early years when relying on external sensors to identify the vehicle maneuvers, Mitrovic took advantage of the acceleration sensor, a circuit board with a gyroscope and the HMM model, to recognize driver behavior, and achieved a better recognition accuracy [6]; however, circuit devices based on external sensors not only increase the hardware costs, but also cause some disturbances to the driver when placed in a specific position (for example, in front of the driver or on the steering wheel).

### 2.3. Based on Smartphone Sensors 

WreckWatch [14] detects traffic accidents using the accelerometer and microphone of a smatphone. With the Dynamic Time Warping (DTW) algorithm, Johnson made full use of a smartphone’s accelerometer, gyroscope and magnetometer to compare the similarity of the sensor waveforms in its MIROAD system, so as to identify vehicle maneuvers [15]. The MIROAD system achieves high accuracy in the recognition of vehicle maneuvers, such as steering, U-turns and lane-changes. Chen estimated the real-time speed of a vehicle through the acceleration sensor and the gyroscope sensor of a mobile phone and judged whether the driver is using the mobile phone while driving based on the different centripetal acceleration of the mobile phone in the driving position and the co-pilot position [11,12]. In research for identifying drunk driving by using smartphone sensors, Dai identified drunk driving with the help of the drunk-driving characteristics [13]. The V-Sense designed by Chen identifies the vehicle maneuvers with a mining algorithm in the time domain which is based on the vehicle’s angular velocity data [16]. V-Sense determines the vehicle maneuvers of a given angular velocity waveform by setting the maximum and the minimum angular velocity, the shortest duration and the minimum waiting time. In addition, the smartphone sensor is also used to monitor road conditions [17,18,19], for example, when the vehicle passes through bumps or potholes on the road, the accelerometer in the smartphone can detect large accelerations perpendicular to the road. These studies confirm the feasibility of using smartphone sensors to sense the dynamics of the vehicle, which can be further used to automatically determine the use of the driver’s telephone.

These works have fully confirmed the feasibility and accuracy of the use of smartphone sensors for identifying vehicle maneuvers, but there are still some shortcomings. For example, MIROAD is based on three sensors (accelerometer, gyroscope and magnetometer), which undoubtedly increases the computational complexity and energy consumption of the mobile phone. When there is a strong magnetic field near the vehicle, the magnetometer will be susceptible to interference, resulting in inaccurate data and an increasing false positive rate. By setting the threshold value of angular velocity in the time domain, the V-Sense can identify the vehicle maneuvers, but due to the differences in the mastery and proficiency of each driver, the vehicle angular velocities will be different, and the vehicle angular velocity may be different even if the same person performs the same driving maneuver several times, which will affect the driving recognition accuracy.

## 3. Vehicle Steering Recognition

### 3.1. Steering Maneuver Modeling

In order to achieve the goal of recognizing dangerous steering maneuvers, we first need to recognize different steering maneuvers. On the real road, the complex and changing traffic conditions also result in a variety of dangerous maneuvers. The dangerous vehicle maneuvers, as defined by the US National Highway Safety Administration (NHTSA) [20], include incorrect signals and lane-changes, as well as frequent and rapid lane-changes. Of course, the dangerous vehicle maneuvers not only include the abovementioned. Several vehicle maneuvers that need to be recognized in this paper are listed in Table 1, where vehicle maneuvers number 1 to number 3 are normal (non-dangerous) vehicle maneuvers, and maneuvers number 4 to number 9 correspond to dangerous vehicle maneuvers.

To identify vehicle steering maneuvers, we focus on the vehicle’s angular velocity, which is characterized by gyroscope data obtained from a smartphone mounted in the vehicle. An existing study [15] verified the feasibility of using smartphone sensors to identify vehicle maneuvers by comparing the mobile sensor data and the vehicle sensor differences between the results of vehicle maneuver data acquisition. 

**Figure 1:**
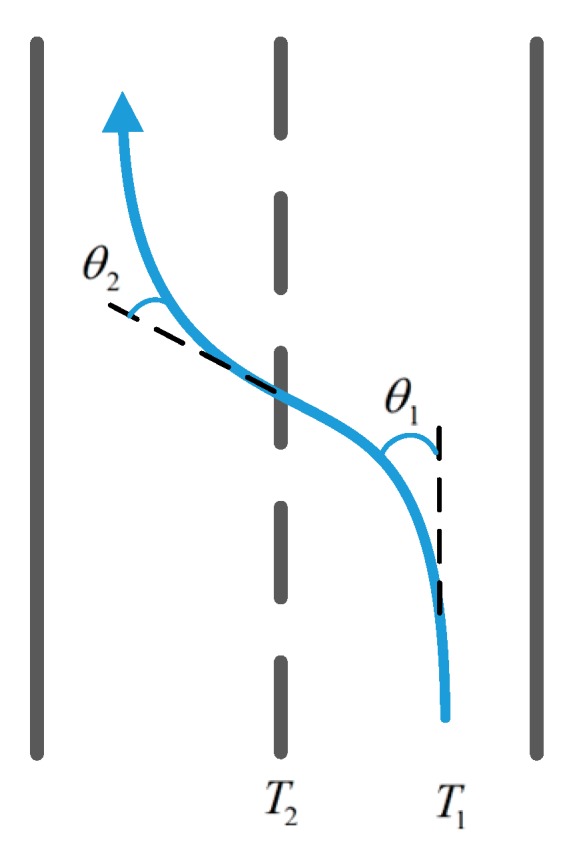
Lane-change from right to left.

As shown in Figure 1, when the vehicle changes lanes from the right to the left, the state of the vehicle during the process of moving can be roughly divided into two parts. First, when the vehicle turns to the left at time $T_{1}$, the direction of turning left and that of the road form an angle $\theta_{1}$, and the gyroscope data shows a positive angular velocity change. Second, when the vehicle turns to the right at time $T_{2}$, the direction of turning right is parallel to the direction of the road, and it forms an angle of $\theta_{2}$ with the direction of the vehicle’s heading and the gyroscope data shows a negative angular velocity change, while the waveform is completely the opposite when the vehicle changes lanes from left to right.

**Figure 2:**
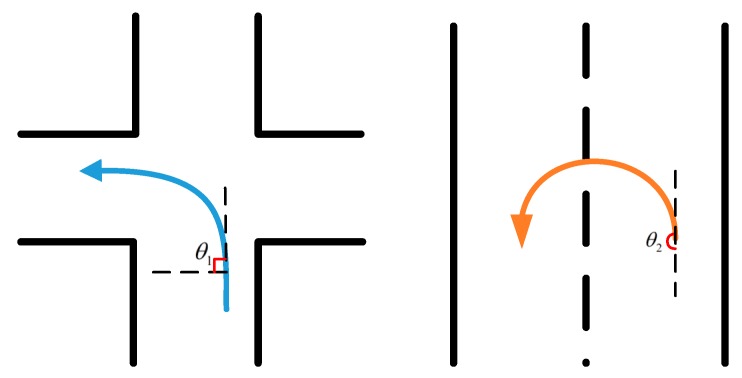
Left turn and left U-turn.

The vehicle turns can be divided into left-turns, right-turns and U-turns. When the vehicle turns to the left, the gyro data shows a positive change in angular velocity, and when turning to the right, it shows just the opposite. In countries with right-hand traffic flow, a U-turn normally means a left-turn. The gyro data is similar to that of turning to the left, as shown in Figure 2.

### 3.2. System Overview

**Figure 3:**
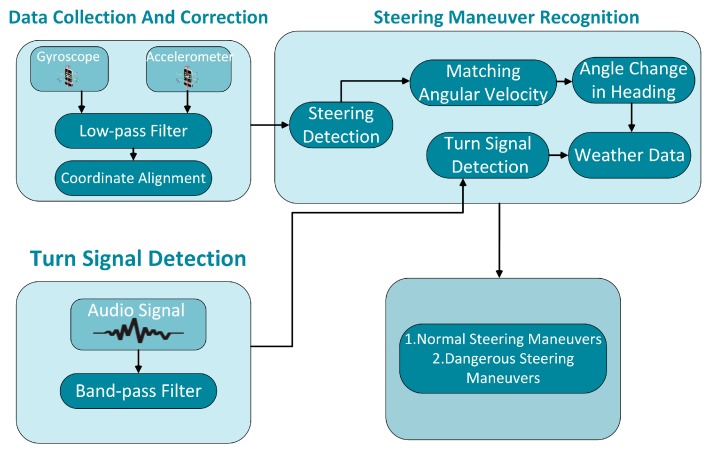
Architecture of the system.

In this section, we describe the architecture of the proposed system aiming at recognizing steering maneuvers described above. As shown in Figure 3, the system consists of three parts as follows:

1. Data Collection and Correction Module: Due to the non-fixed location of the smartphone in the vehicle, the inertial sensor data can’t directly reflect the angular velocity information when the angle of the vehicle’s body changes. We obtain the correct angular velocity by aligning the three-axis gyroscope coordinate system with the vehicle’s coordinate system with the help of the accelerated velocity data.
2. Turn Signal Detection Module: Many traffic accidents are caused by drivers who don’t use the signal lights when turning or changing lanes. Therefore, we use the microphone sensor to determine whether the driver turned on the turn signal by detecting the audio of the turn signal.
3. Steering Recognition Module: The current recognition algorithm for vehicle maneuvers is not comprehensive enough. Therefore, in this part, we first design a reasonable endpoint detection algorithm to determine the occurrence of steering maneuvers. Then we get the recognition algorithm based on waveform matching of the vehicle’s angular velocity, steering angle of the vehicle’s heading, the audio frequency of turn signals and weather conditions.

### 3.3. Data Collection and Correction

The inertial measurement unit (IMU), also called the inertial sensor, is a three-axis gyroscope and a three-axis accelerometer. The gyroscope sensor is used to measure the angular movement of a smartphone. The accelerometer sensor is used to measure the acceleration of a smartphone. If the smartphone is placed in a vehicle and remains motionless, the inertial sensor can collect the information of the vehicle’s movement in the three-dimensional space, but due to the unfixed state of the smartphone in the vehicle, the gyroscope data can’t directly reflect the angular velocity of the vehicle, so this section mainly focuses on the data collection and correction of the inertial sensor. Firstly, we use low-pass filter to filter out high-frequency noise, then the acceleration data is used to align the gyroscope with the vehicle’s coordinate system. Finally, the gyroscope data is the angular velocity data when the vehicle changes its angle.

#### 3.3.1. Data Collection of the Inertial Sensor

Sampling rate has a direct impact on the recognition of steering maneuvers. If the sampling rate is too low, it will cause signal distortion, while if the sample rate is too high, it will increase the power consumption and memory footprint of the smartphones. Given that the duration of a certain steering maneuver of a vehicle is 2 s [2], the complete waveform cycle recorded by the gyroscope is 2 s and the waveform frequency is 1/2 Hz. According to the Nyquist sampling theorem, when the sampling frequency is twice as high as that of the signal, the digital signal after sampling can retain the information of the original signal. In real-world applications, we need to ensure that the sampling frequency is five to ten times as much as the highest frequency of the signal. To ensure that the signals collected by the gyroscope can completely restore the information of vehicle’s steering maneuver, we will set the sampling rate as 20 Hz by allowing for the consumption of smartphone’s battery and memory at the same time.

#### 3.3.2. Data Filtering of the Inertial Sensor

**Figure 4:**
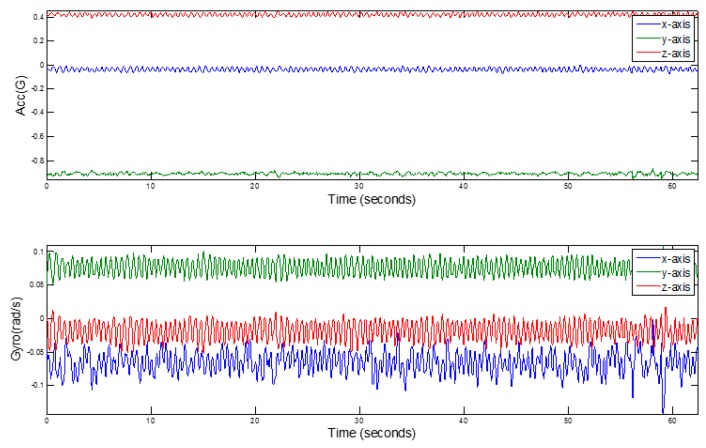
The raw gyroscope and accelerometer data.

In the course of driving, due to the conditions of the road and the vehicle itself, there will be some high-frequency noise interfering with the inertial sensor data. Figure 4 shows the data collected by the three-axis gyroscope and the accelerometer of the smartphone in the car.

**Figure 5:**
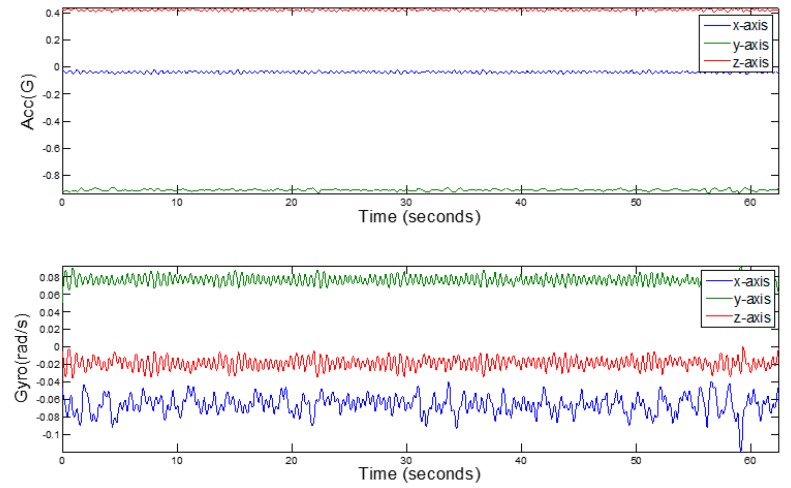
The filtered gyroscope and accelerometer data.

To avoid the interference from the noise on the data collection, we use a Butterworth low-pass filter to filter out and block any high-frequency noise, so as to obtain an effective signal. The filtering effect is shown in Figure 5.

#### 3.3.3. Coordinate Alignment

**Figure 6:**
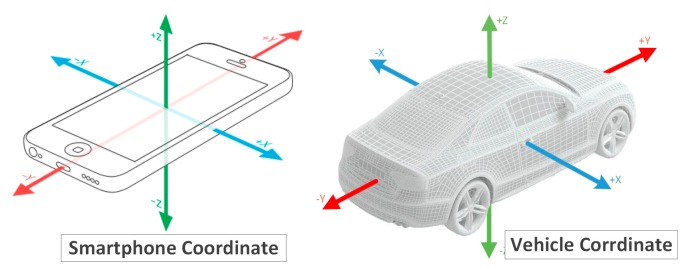
The coordinate systems of a smartphone and a vehicle.

A smartphone’s coordinate system actually describes the coordinate system of its internal inertial sensor. Therefore, the alignment of the gyro’s coordinate system and the vehicle’s coordinate system is equivalent to that of the smartphone’s coordinate system and vehicle’s coordinate system. As shown in the left subfigure of Figure 6, the standard coordinate system of a smartphone is designed with the screen set as a reference object, when the smartphone is motionless. When the smartphone is in a horizontal situation, the x-axis represents the horizontal direction from left to right, the y-axis represents the vertical direction from bottom to top, and z-axis represents the direction perpendicular to the screen from the inside out, relative to the screen surface. When the smartphone rotates, the three-axis can change as the direction of the screen changes. The three-axis data follows the right-hand rule. The right subfigure of Figure 6 shows the vehicle’s coordinate system, which is similar to the smartphone’s coordinate system, and can be also called a three-axis coordinate system. Because vehicles often drive on a horizontal road, the z-axis is aligned with the direction of gravitational acceleration of the Earth coordinate system, and the x-axis and the y-axis of the vehicle coordinate system and the x-axes and y-axes of the Earth coordinate system are at an angle. The vehicle coordinate system is aligned with the Earth coordinate system when the vehicle is moving toward the geomagnetic north.

In the process of driving, the driver’s smartphone is generally placed in the vehicle’s bracket or a groove of the instrument panel. The z-axis of the vehicle’s coordinate system is always aligned with the direction of gravitational acceleration (the slope will temporarily not be considered). Therefore, when the smartphone is in an uncertain position of the vehicle, the data recorded by the three axes of the gyroscope often is the sub-vector data [21] distributed in that three axes, which can’t visually reflect the changes of vehicle’s angular velocity. Therefor we need to align the smartphone’s coordinate system with the vehicle’s one.

Before studying the method of alignment of coordinate systems, we briefly explain the concept of coordinate rotation. In the Cartesian coordinate system, we let a unit vector (1,0,0) along the x-axis multiply by a 3 × 3 rotation matrix, and then we get the first column of elements of the matrix. Similarly, when the unit vectors of y-axis and z-axis multiply the rotation matrix, respectively, the results are the elements in the second and third column, respectively. Besides, each column of the rotation matrix is expressed as the fixed axis after rotation. For example, the first column means rotating around the x-axis, the second column means rotating around the y-axis, while the third column means rotating around the z-axis.

**Figure 7:**
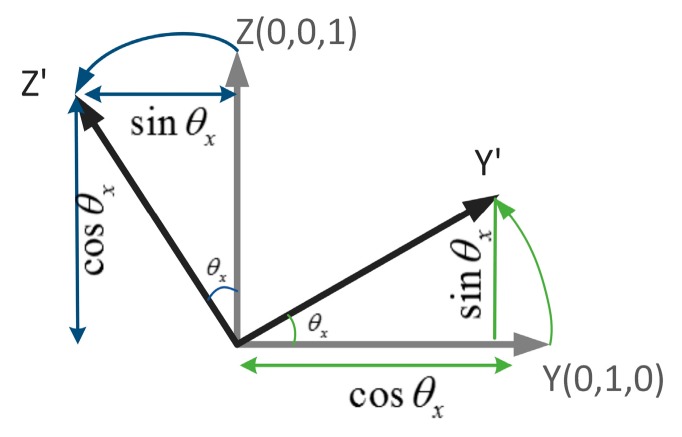
Rotation around the x-axis.

As shown in Figure 7, a coordinate system unit rotates around the x-axis. The initial situations of the y-axis and the z-axis are (0,1,0) and (0,0,1), respectively. When the rotation angle is $\theta_{x}$, the y-axis changes from (0,1,0) to $\left(0,\cos\theta_{x},\sin\theta_{x}\right)$ Similarly, the z-axis also changes from (0,0,1) to $\left(0,-\sin\theta_{x},\cos\theta_{x}\right)$. The changeable y and z axes can be treated as the column vectors of the 3 × 3 rotation matrix, so the rotation matrix can be expressed as follows:

(1) $$R_{x}\left(\theta_{x}\right)=\left(\begin{array}{ccc} 1 & 0 & 0\\ 0 & \cos\theta_{x} & -\sin\theta_{x}\\ 0 & \sin\theta_{x} & \cos\theta_{x} \end{array}\right)$$

**Figure 8:**
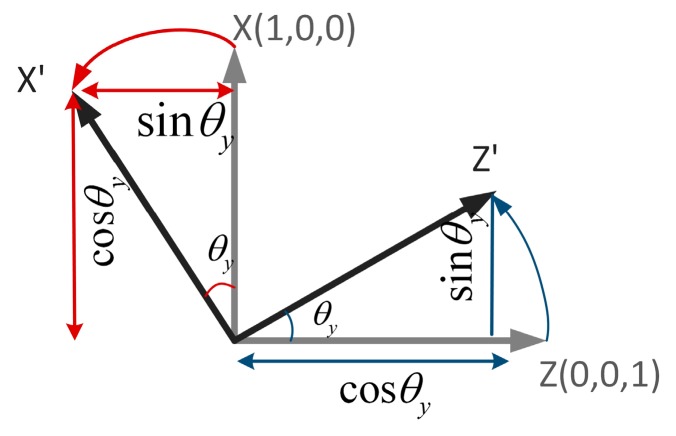
Rotation around the y-axis.

Similarly, as shown in Figure 8, when a unit of the coordinate system rotates around the y-axis, the initial positions of the x-axis and the z-axis are (1, 0, 0) and (0, 0, 1), respectively. When the rotation angle is $\theta_{y}$, the x-axis changes from (1,0,0) to $\left(\cos\theta_{y},0,-\sin\theta_{y}\right)$. Similarly, the z-axis also changes from (0,0,1) to $\left(\sin\theta_{y},0,\cos\theta_{y}\right)$. The changeable x and z axes can be treated as the column vectors of the 3 × 3 rotation matrix, so the rotation matrix can be written as:

(2) $$R_{y}(\theta_{y})=\left(\begin{array}{ccc} \cos\theta_{y} & 0 & \sin\theta_{y}\\ 0 & 1 & 0\\ -\sin\theta_{y} & 0 & \cos\theta_{y} \end{array}\right)$$

Finally, when the axis rotates around the z-axis, the rotation matrix is as follows:

(3) $$R_{z}(\theta_{z})=\left(\begin{array}{ccc} \cos\theta_{z} & -\sin\theta_{z} & 0\\ \sin\theta_{z} & \cos\theta_{z} & 0\\ 0 & 0 & 1 \end{array}\right)$$

Equations (1)–(3) are the rotation matrices in which the three-axis coordinate system rotates around a fixed axis. Any rotation of the coordinate system can be disassembled into the above three kinds of combinations rotating around the fixed axis, but the sequence of rotation around the fixed axis can’t be changed. For example, the coordinate system rotates around the z, y and x-axes, and the attitude A rotates to the attitude B, so the rotation matrix R is:

(4) $$R=R_{z}(\theta_{z})R_{y}(\theta_{y})R_{x}(\theta_{x})$$

After the matrix inversion, we can also achieve the goal of rotating the attitude B to the attitude A in turn, then the rotation matrix $R^{\prime }$ is:

(5) $$R^{\prime }=R_{z}{\left(\theta_{z}\right)}^{T}R_{y}{\left(\theta_{y}\right)}^{T}R_{x}{\left(\theta_{x}\right)}^{T}$$

**Figure 9:**
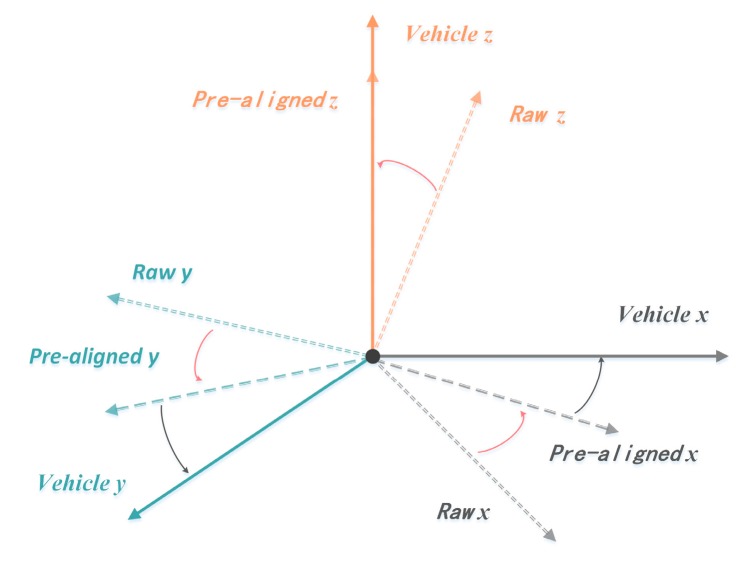
Coordinate alignment.

Therefore, the alignment of the coordinate system of this paper is based on the rotation of the coordinate system, namely, the smartphone aligns with the vehicle’s coordinate system after the rotation. How to know the angle between the current posture of the smartphone and the post-rotation posture has become an urgent problem that needs to be solved. Fortunately, most of the current smart phones provide this kind of data interface. For example, the Rotation Matrix [22] of the Apple iPhone can provide the rotation matrix from the reference coordinate system to the current position. Given that the reference coordinate system is the alignment between the smartphone’s z-axis and the direction of gravity, and x-axis and y-axis are in any directions of the horizontal plane. Therefore, the alignment of the coordinate system will be divided into two parts:

1. The first rotation: at first, rotating the smartphone from the current posture to the reference coordinate system through the Inverse Matrix of the Rotation Matrix. Assuming the original reading of the accelerometer as a, while the original reading of three-axis gyroscope as w, then we can obtain the acceleration and angular velocity as shown in Figure 2c after the first rotation:
  (6) $$\begin{array}{l} {\hat{a}}_{1}=R_{x}{\left(\theta_{x}\right)}^{T}R_{y}{\left(\theta_{y}\right)}^{T}R_{z}{\left(\theta_{z}\right)}^{T}\cdot a\\ {\hat{\omega }}_{1}=R_{x}{\left(\theta_{x}\right)}^{T}R_{y}{\left(\theta_{y}\right)}^{T}R_{z}{\left(\theta_{z}\right)}^{T}\cdot \omega \end{array}$$
2. With the second rotation: we can achieve the goal of the second alignment by obtaining the angle between the x, y-axis of the smartphone’s coordinate system and the vehicle’s. The process of rotation is shown in Figure 9.

In the second rotation, we need to get the angle between the x, y-axis of the smartphone’s coordinate system and the vehicle’s, so as to get the rotation matrix around the z-axis. Therefore, we assume that there is a known acceleration. When the vehicle is accelerating, the direction of acceleration is along the vehicle’s driving direction. The acceleration has less influence on the side of the vehicle, namely, we can think that the acceleration at this time is increasing along with the vehicle’s y-axis. We found that the vehicle satisfies this assumption when it’s accelerating or at startup. Therefore, after the initial rotation, we can use the acceleration of the vehicle to calculate its mobile phone coordinate system x, y-axis component angle and then the second rotation. As shown [21]:

(7) $$\theta =-a\tan2\left(\vec{{\hat{a}}_{1y}},\vec{{\hat{a}}_{1x}}\right)$$

In that equation, $\vec{{\hat{a}}_{1y}},\vec{{\hat{a}}_{1x}}$ means the components’ average value of the accelerometer in the x-axis and y-axis of the smartphone’s coordinate system after the first rotation. atan2() is an inverse trigonometric function, and we can determine the quadrant of the angle from the two input values. Therefore, we can get the rotation matrix which rotates θ degrees around the z-axis of the smartphone’s coordinate system:

(8) $${R^{\prime }}_{z}(\theta )=\left(\begin{array}{ccc} \cos\theta & -\sin\theta & 0\\ \sin\theta & \cos\theta & 0\\ 0 & 0 & 1 \end{array}\right)$$

According to the angular velocity, we can obtain the rotation matrix after the rotation around the z-axis:

(9) $${\hat{\omega }}_{2}={R^{\prime }}_{z}(\theta )\cdot {\hat{\omega }}_{1}$$

${\hat{\omega }}_{2}$ denotes the angular velocity after the second rotation, namely, the vehicle’s steering angular velocity. In the smartphone’s interface, we can set the smartphone’s coordinate system at this time as the reference coordinate system. Hereafter, during the data collection process, an alignment of reference frames can be completed according to the reverse rotation of the rotation matrix in the smartphone’s interface, so as to eliminate the trouble of completing the second alignment.

### 3.4. Turn Signal Detection

Drivers need to use the turn signals when the vehicle turns, changes lanes or makes U-turns, otherwise these behaviors are considered dangerous vehicle maneuvers. In order to detect whether the driver has turned on the turn signal, we take the following steps: (1) using the smartphone’s microphone to collect sound samples when the vehicle’s turn signal has been turned on; (2) using a z band-pass filter to extract the signal of the turn signal use; (3) combining the threshold of audio amplitude from the turn signal and the threshold of time interval, so as to determine whether the turn signal is turned on.

#### 3.4.1. The Features of Turn Signals

**Figure 10:**
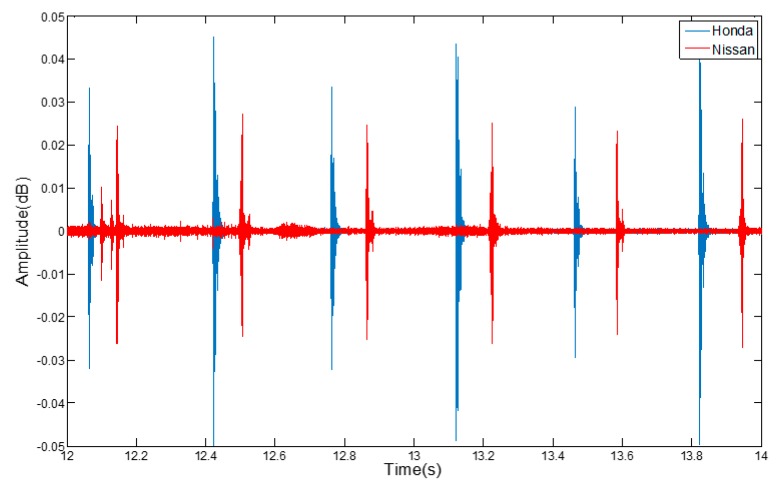
The features of turn signals.

By recording the audio signal of a turn signal, we can find that the sound of modern vehicles is a regular signal with a fixed frequency. As shown in Figure 10, the waveform reflects that the audio amplitude of the turn signal increases and decreases in a regular way, while its cycle remains consistent. The audio amplitude is about 0.36 dB, which is similar to a kind of “Pata” sound as heard by the human ear.

#### 3.4.2. Collect the Audio Signal

First of all, two different vehicles are used to collect the audio signals from the turn signals without interference. The two vehicle models were a Nissan Novelty and a Honda Si Core Rui. In order to ensure the authenticity of the experimental environment, we collected the audio signal from the turn signal while people were chatting and music was played on the speakers in the vehicle.

#### 3.4.3. Extract the Audio Signal

**Figure 11:**
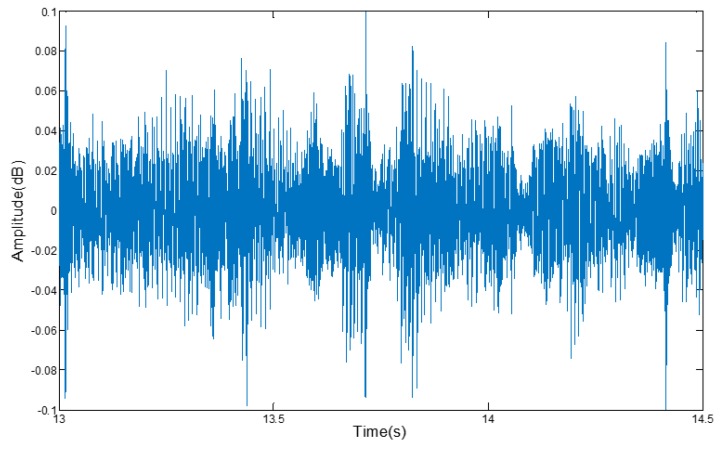
The amplitude of the audio signal with noise.

In the driving process, noise from the environment will interfere with the smartphone’s microphone to judge whether the turn light is turned on. That noise may come from people talking inside the vehicle, the vehicle speakers, the noisy environment around the road, and the internal noise of driving. As shown in Figure 11, due to the interference of music from the vehicle’s audio and chatting voices, the sound signal from turn signals recorded by the smartphone lost its characteristic profile. 

**Figure 12:**
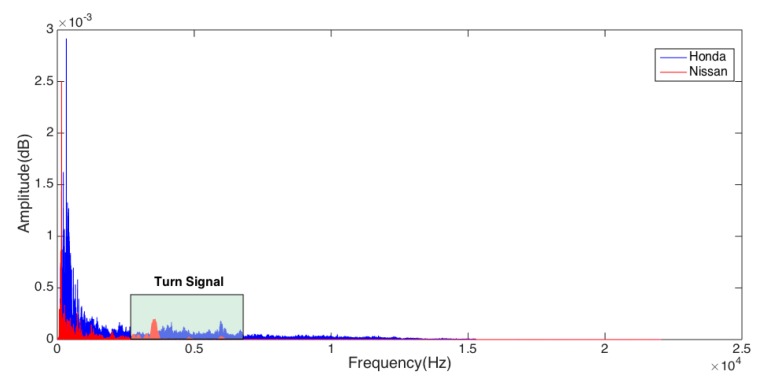
The spectrum of the audio signal with noise.

With the help of a lot of experiments, we found that the audio signal of the turn signal is cyclical and its amplitude never changes, and the frequency of turn signals is mainly distributed between 3400 Hz~10,000 Hz. As shown in Figure 12, the voice frequency ranges from 300 Hz to 3300 Hz when talking with a normal voice, while the internal noise of the vehicle is mainly from the external noise entering through the air or through the shaking of the car body, and the frequency of these noises ranges from between 125 Hz~1200 Hz [23], so we can use a band-pass filter to extract the turn signal. 

**Figure 13:**
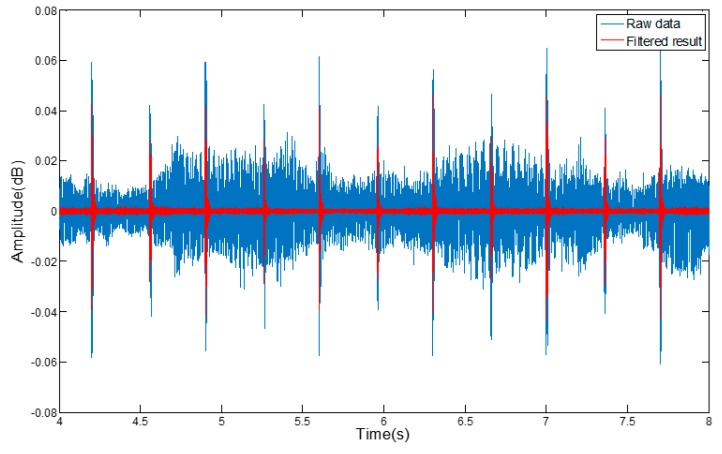
The amplitude of the audio signal after filtering.

Eventually, after the noise reduction processing, the audio signal from the turn signal is shown in Figure 13.

#### 3.4.4. Turn Signal Detection

With the help of a large number of experiments, we found that the threshold value of the turn signal’s amplitude $A_{t}$ is 0.04 dB. In order to avoid mistaking the noise for the audio signal from the turn signal, we added the threshold of time interval of $T_{t}$, which is greater than the waveform period of 0.05 s. Therefore, it is possible to determine whether or not the driver turned on the turn signal when the thresholds $A_{t}$ and $T_{t}$ are satisfied.

### 3.5. Vehicle Steering Recognition

**Figure 14:**
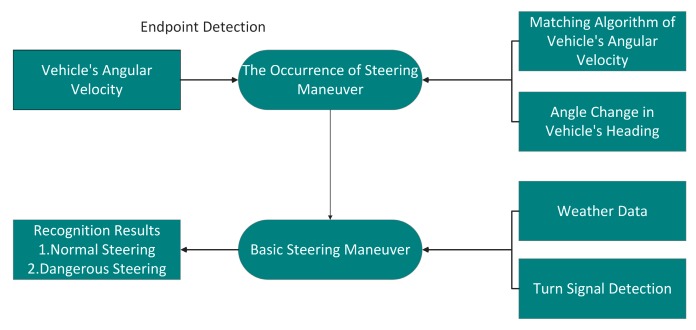
Vehicle steering recognition.

At present, some vehicle steering maneuver recognition methods adopt a single vehicle’s angular rate matching method and jointly use a multi-sensor system for recognition. Based on the time domain characteristics of vehicle angular velocity, other methods perform data mining to recognize the driving behavior. All of these methods not only increase the computational complexity and the misjudgment rate, but also recognize the driving behaviors without a comprehensive consideration. The main purpose of this paper was to recognize the different steering maneuvers. However, without sufficient characterization, the vehicle angular velocity data can’t distinguish between vehicle turns and U-turns. Meanwhile, there are various conditions for measuring dangerous vehicle maneuvers. For example, in severe weather conditions (such as rainy or snowy weather), some normal vehicle maneuvers can also lead to traffic accidents on the slippery roads. In this paper, we combined the matching of angular velocity, steering angle of a vehicle’s heading, weather factors and the detection of the vehicle’s turn signals together to recognize the vehicle maneuvers, and we also designed a reasonable endpoint detection algorithm to determine the occurrence of particular steering maneuvers. As shown in Figure 14, we first determine the occurrence of steering maneuvers through an endpoint detection algorithm. Then the turns, lane changes and the U-turns of the vehicle are preliminarily distinguished according to the matching of the vehicle angular velocity and the steering angle of the vehicle’s head. Finally, by setting the threshold of the angular velocity based on different weather conditions and using the result of whether the turn signals are turned on, we can further determine whether the steering maneuver is dangerous driving or not.

#### 3.5.1. Steering Maneuver Detection

In order to detect the beginning and the end of different vehicle maneuvers (such as steering and U-turns), the starting point and end point of the waveform of the vehicle’s angular velocity are determined by the endpoint detection method. The endpoint detection method [24,25] mainly takes advantage of the time-domain characteristic parameters of signals, such as short-time energy, zero-crossing rate, correlation and so on. In the time domain, the common endpoint detection method is often determined on the basis of several of the parameters proposed above. In this paper, we adopt the endpoint detection scheme based on the parameters of the short-term energy spectrum. With this method, it is easy to distinguish the effective signal segment from noise when the Signal to Noise Ratio (SNR) is very high. Now the short-term energy will be introduced as follows:

Short-term energy: the main difference between signal and noise lies in their energy. Signal energy equals the sum of the superposition of the effective signal energy and the noise segment, and the energy of the signal segment is more than that of the noise segment [24,25]. The energy of the signal changes obviously along with time, and for the sequence {x(m)}, the energy is defined as:
  (10) $$E=\sum_{m=-\infty }^{\infty }x^{2}(m)$$

Equation (10) shows the energy of an infinitely long signal, and such a long-term energy accumulation can’t help to distinguish the signal from the noise energy. We usually intercept a time slice from the signal, so as to analyze and process that signal. Therefore, we can use different truncated functions to intercept signals, and the truncated function is also known as the window function. In the calculation of short-term energy, by relying on the sliding window function to accumulate the computational energy, we can obtain a reliable short-term energy spectrum. We use the rectangular window function in this paper with its formula as follows. *N* of that equation denotes the window length:

(11) $$W_{R}=\left\{ \begin{array}{lll} 1 & & \left(0\leq n<N-1\right)\\ 0 & & \left(\mathrm{other}\right) \end{array}\right.$$

Then the signal variation of the gyroscope corresponding to a vehicle maneuver is:

(12) $$x_{n}(m)=x\left(m\right)w\left(n-m\right),n-N+1\leq m\leq n$$

w(n−m)denotes the window function, *N* is the window’s length, and the value ranges of *n* are 0, *T*, *2T*, ..., finally, *T* is the sliding distance of the window. Therefore, the short-term energy spectrum of the vehicle’s angular velocity corresponding with a vehicle maneuver can be expressed as:

(13) $$E_{n}=\sum_{m=n-N+1}^{n}{\left[x\left(m\right)w\left(n-m\right)\right]}^{2}$$

We need to set the high energy threshold of $E_{high}$ and the low energy threshold of $E_{low}$ before the testing. The short-term energy spectrum of the vehicle’s angular velocity is divided into three parts: static segment, transition segment and vehicle maneuver segment. If the energy value exceeds the low energy threshold in the stationary segment, it is considered to have entered a transition section. In the transition section, if the energy spectrum can’t reach the high energy threshold, we can’t judge whether it has entered the vehicle maneuver section, and if the energy value is below the low energy threshold, it will come back to the stationary segment. When the energy value exceeds the high energy threshold, we need to introduce a minimum time interval $T_{short}$ to distinguish between noise and real vehicle maneuvers. Namely, when the energy duration is less than the minimum time interval, it is considered to be noise and comes back to the stationary section, but when the energy duration is greater than the minimum time interval, it is considered to have entered the vehicle maneuver section. After recording the energy duration and marking the start point and the end point, the program will be back to the static section, waiting for the arrival of the next waveform. Now, we will analyze the effectiveness of the endpoint detection method in this paper based on the vehicle’s angular velocity waveforms.

**Figure 15:**
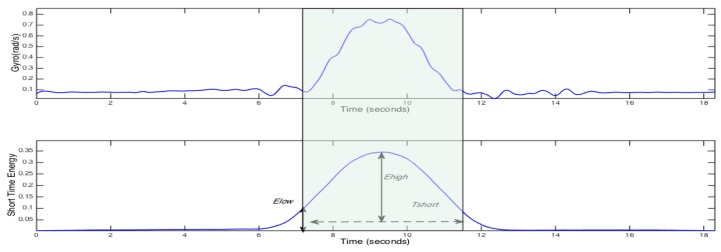
Gyro and short time energy for a left lane change.

The waveforms of a vehicle’s angular velocity and its short-term energy spectrum are shown in Figure 15. By observing the short-term energy spectrum, we can clearly see that the noise and effective signal are distinguished and we can accurately distinguish between the start and end points of the waveforms by setting the high-energy threshold of $E_{high}$ and the low energy threshold of $E_{low}$, and the shortest duration of $T_{short}$.

**Figure 16:**
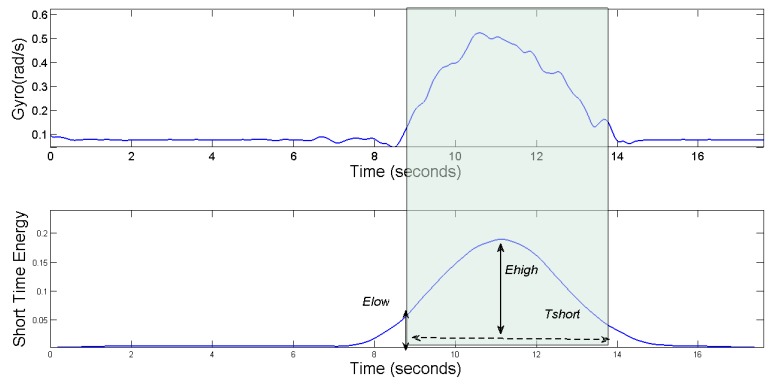
Gyro and short time energy during a left turn.

Figure 16 shows the waveforms of the vehicle’s angular velocity of a left-turn driving maneuver, and its energy spectrum. In the rectangle frame of that figure, we can get the starting point and end point of the steering driving behaviors by the endpoint detection method. From the waveforms of the vehicle’s angular velocity, we can see that the angular velocity is no more than 0.5 rad/s during the vehicle’s normal steering, with a duration of 4 s. 

**Figure 17:**
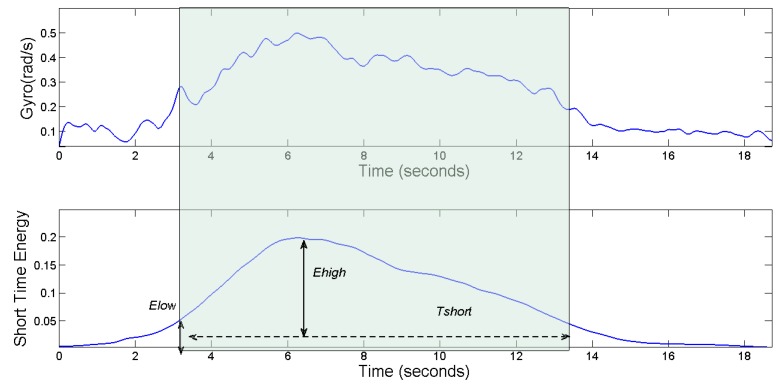
Gyro and short time energy during a U-turn.

Figure 17 shows the waveforms of the vehicle’s angular velocity of a U-turn driving vehicle, and its energy spectrum. We can see that the peak value of vehicle’s angular velocity in the U-turn is close to that of the left-turn driving, but the duration of the angular velocity is more than 8 s during the U-turn, which takes longer than a left-turn. In a left-side-of-the-road driving country, the vehicle turns to the right to make a U-turn. Therefore, the waveform of the gyroscope is similar to that of the right-turn waveform.

The vehicle’s angular velocity data recorded by the gyroscope can clearly express the steering maneuvers, and its short-term energy spectrum can accurately reflect the steering maneuvers. All of these prove the feasibility of using the gyroscope to identify steering maneuvers, and the validity of the endpoint detection method we designed in this paper.

#### 3.5.2. Matching Vehicle’s Angular Velocity

After the analysis above, we can find that the waveform of a vehicle’s angular velocity can clearly reflect its steering maneuvers. What we need to do is match and recognize the data of the vehicle’s angular velocity, then, we can initially distinguish the steering maneuvers such as turns, lane-changes and U-turns. Because the vehicle angular velocity is a kind of time series, at present, the research ideas about time series recognition are divided into two kinds: (1) using data mining technology to extract features in the time sequence, so as to recognize the vehicle maneuvers; (2) recognizing vehicle maneuvers on the basis of common speech recognition algorithms. In the recognition of vehicle maneuvers, the data of gyroscopes and speech signals have some similarities; different drivers have different driving habits and proficiency, and with the different road conditions and other reasons, the length of the duration of the data of gyroscope will be different. Therefore, in the recognition of vehicle maneuvers, we consider the use of dynamic time wrapping (DTW) algorithm.

**Figure 18:**
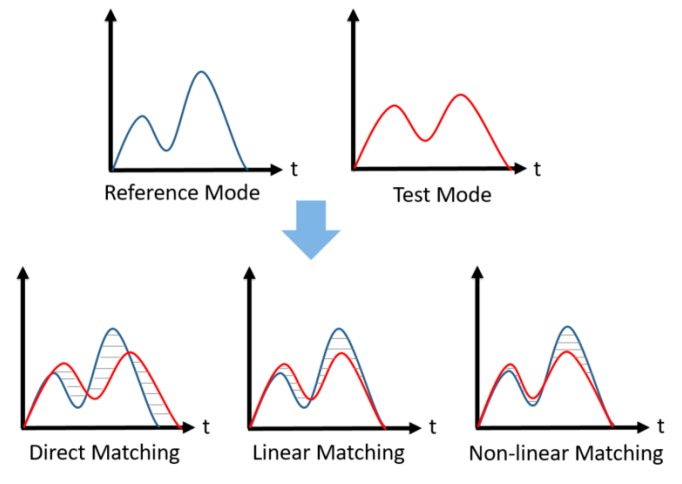
Three matching modes.

The DTW algorithm is a method to measure the similarity of two different time series [26,27], and it is often used in isolated speech recognition, gesture recognition and information retrieval. As shown in Figure 18 the two times series of different lengths, in terms of direct matching or linear matching method to calculate the Euclidean distance will lead to a significant error (shaded), DTW minimizes the matching error by extending or shrinking the time series nonlinearly and then calculating the similarity between the two time series [28]. 

**Figure 19:**
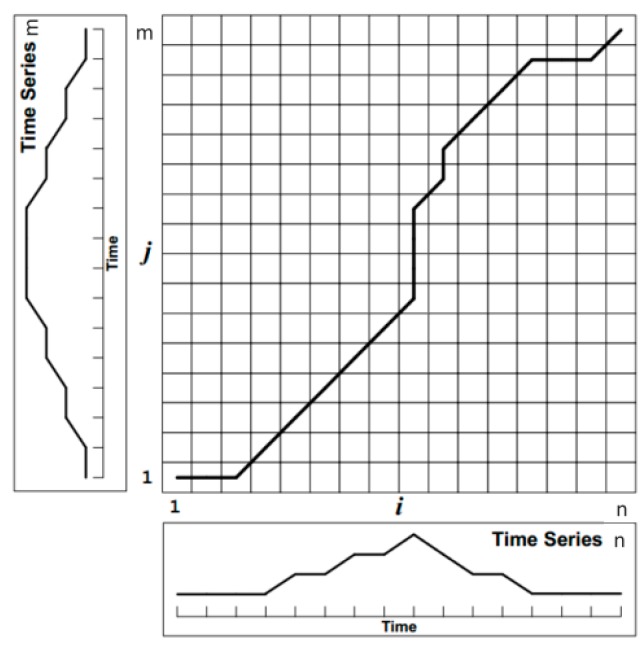
The DTW principle.

Let the time series $R=r_{1},r_{2}\ldots ,r_{i},\ldots ,r_{m}$ be the reference template sequence, the time series $T=t_{1},t_{2}\ldots ,t_{j}\ldots ,t_{n}$ is the template sequence to be tested, and m≠n. The principle of the algorithm is shown in Figure 19. 

The matrix in Figure 19 is a m×n matrix, the y-axis is m elements of the reference template, the x-axis is the n elements of the template to be tested. The matrix elements (i,j) represent the Euclidean distance between $r_{i}$ and $t_{j}$:

(14) $$d\left(r_{i},t_{j}\right)=\sqrt{\overset{n}{\underset{i=1,j=1}{\sum }}{\left(r_{i}-t_{j}\right)}^{2}}$$

The DTW algorithm first computes the distance between the elements of the two sequences, finds the matching distance matrix, and then searches for a path where the cumulative distance is the smallest, and defines the path W as:

(15) $$W=w_{1},w_{1},\ldots ,w_{k},\ldots w_{N}\max\left(m,n\right)\leq N\leq m+n-1$$

In searching for the path *W*, the following constraint conditions must be satisfied, otherwise the searched path may make the similarity of two fundamentally different time series high.

1. Boundary Constraints: The search must occur between the start and end points, since the order of the time series does not change, therefore, in Figure 19 it appears from the lower left corner to the upper right corner of the end, which is:
  (16) $$\{\begin{array}{c} w_{1}=(1,1)\\ w_{N}=(m,n) \end{array}$$
2. Continuity Constraints: in the search process, it can’t cross a certain element to match, so as to ensure that the reference template R and the template T to be tested for each coordinate in *W* appear. Suppose $w_{k-1}=\left(a^{\prime },b^{\prime }\right),\;w_{k}=\left(a,b\right)$, need to meet:
  (17) $$\{\begin{array}{c} a-a^{\prime }\geq 0\\ b-b^{\prime }\geq 0 \end{array}$$
3. Monotonic Constraint: To ensure that the search process is monotonically increasing with time. Suppose $w_{k-1}=\left(a^{\prime },b^{\prime }\right),w_{k}=\left(a,b\right)$, need to meet Equation (17).

After the above three constraints, the process of searching for the best path can be described as follows: the path of each point in the template matrix has only three directions, for example, the path has passed the point (i,j), then its previous grid point can only be (i−1,j),(i,j−1),(i−1,j−1), and finally get their regularization of the shortest cumulative distance. We define the cumulative distance as shown below:

(18) $$D\left(i,j\right)=Dist\left(r_{i},t_{j}\right)+min\left\{D\left(i-1,j-1\right),D\left(i-1,j\right),D(i,j-1)\right\}$$

The calculated distances of all points in the template matrix are summed up according to the abovementioned constraints from (0,0), and when the end point (*m*,*n*) is reached; the smaller the cumulative distance is, the higher the similarity is, so the time complexity of the DTW algorithm is O(mn).

In the recognition of driving behavior, the gyroscope data and the voice signals have some similarities. For example, different driving habits, proficiency and road surface during the driving process can cause the gyroscope data duration to be different. Therefore, in the recognition of driving behavior, we consider the use of a dynamic time regularization algorithm. However, in the face of a number of vehicle maneuvers data for a long time, the high time complexity of the DTW algorithm will affect the efficiency of the system. How to reduce the time complexity of DTW algorithm will be the problem we need to solve in the system design.

**Figure 20:**
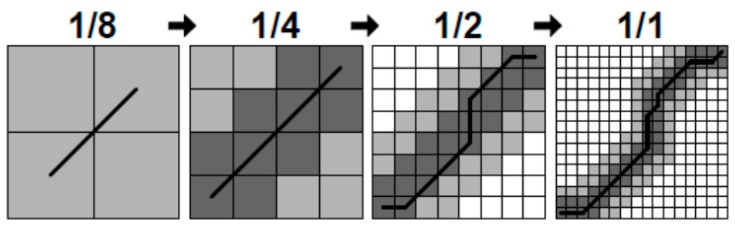
FastDTW principle.

The conventional DTW uses the idea of dynamic programming to compute the similarity of two time series. Let m,n be the length of the reference sequence template and the sequence template to be measured respectively. If *m* = *n*, then the DTW algorithm calculates the optimal routing path corresponding to the time and space complexity of $O\left(n^{2}\right)$. The traditional DTW algorithm has a high time complexity, and when the system stores a large amount of driving behavior data, the efficiency of the algorithm is obviously slowed down. To this end we need to improve the DTW algorithm to improve its operating efficiency. Common DTW algorithm acceleration methods include limiting the path search width, data abstraction and indexing [29,30,31]. We will use the FastDTW algorithm proposed by Salvador [32], which the core idea of improved DTW algorithm draws on both limiting search width and data abstraction. The algorithm is divided into three steps:

(1) Reduce the resolution: contraction time series as far as possible with smaller data points to represent the original time series, so that one can reduce the dimension of the template matrix, that is to reduce the resolution.
(2) Projection: Perform a DTW algorithm on a lower-resolution template matrix. The optimal path is searched for.
(3) Increasing resolution: The template matrix cells that pass through the regular path, obtained at low resolution, are further refined to a higher resolution on the template matrix. As shown in Figure 20, in order to find the optimal regular path, we need to add search radius *r* at higher resolution, that is to extend K cells to both sides of the regular path.

Due to the limitations of the search radius, the FastDTW algorithm is actually only the shadow of the above part of the cell search. In Figure 20, from the low resolution to the high resolution, the FastDTW algorithm calculates 4 + 16 + 44 + 100 = 164 cells, whereas the traditional DTW algorithm calculates $16^{2}=256$ cells. Search radius r also determines the accuracy of FastDTW, when r increases, FastDTW can search for more cells to improve the accuracy of the algorithm itself, but it also increases the time and space overhead, when r is reduced, the FastDTW algorithm reduces the time and space complexity, but the accuracy will decrease, so the choice of parameter r will determine the efficiency of FastDTW algorithm. Normally, r is set to 1 or 2.

**Figure 21:**
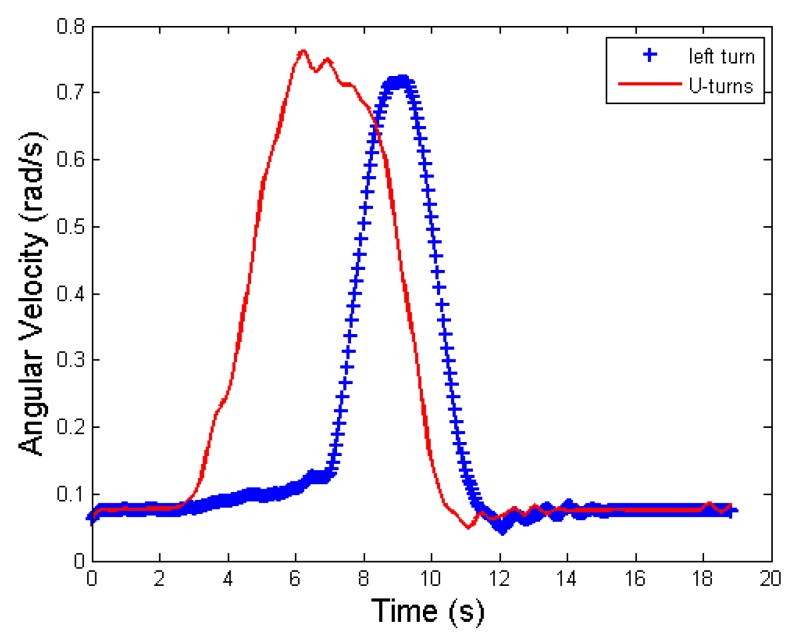
Angular velocity of left turn and U-turn.

To sum up, we chose the FastDTW algorithm for vehicle angular velocity matching recognition. However, due to the different road conditions, different vehicles, and driver’s different driving habits, the waveforms of the vehicle’s angular velocity from the left-turn and U-turn will be different, as shown in Figure 21.

We can see the two waveforms, whose amplitudes are more than 0.7 rad/s, are similar. The waveforms of the duration of the U-turn are not much higher than those of the left-turn. If the waveform of a U-turn is more similar with that of a left-turn, the matching error rate of FastDTW algorithm will increase quickly. In order to distinguish the left-turn and the U-turn, we added the criterion on the basis of recognizing the similarity between the two waveforms, namely, to further distinguish the left-turn and the U-turn by the turning angle of the vehicle’s heading.

#### 3.5.3. Heading Angle Change 

The steering angle of the vehicle’s heading is about 90° before and after a left-turn, and it is about 180° before and after a U-turn of the vehicle. The steering angle of the vehicle’s heading will help us to distinguish between a left-turn and a U-turn. The MIROAD system makes use of the magnetometer (compass) in the smartphone’s sensor to obtain the observed data from the steering angle of the vehicle’s heading. However, the magnetometer is subject to environmental effects, resulting in some errors in the magnetometer’s readings if it is in a strong magnetic field. Besides, if the angle of the vehicle’s heading changes very fast, the magnetometer can’t quickly sense the steering angle of the vehicle’s heading, which will not allow us to get accurate data. In this paper, we use the data of vehicle’s angular velocity to calculate the vehicle’s heading steering angle. Given the vehicle’s angular velocity as ‘g’, then the steering angle of vehicle’s heading is expressed as follows:

(19) $$\theta =\theta_{0}+\sum_{i=0}^{n}g_{i}\cdot \Delta t$$

Formula $g_{i}$ denotes the vehicle’s angular velocity at any time, while Δt is the interval of sampling time. Since we found that some errors may exist between θ of the steering angle of a vehicle’s heading in a practical situation, we set the range of change between the vehicle’s left-turn and U-turn. When the obtained value of θ, the small angle of vehicle, falls in one of the intervals, we can determine which vehicle maneuver it belongs to. Assuming that the vehicle maneuver ‘*a* = 0’ stands for a turn, and ‘*a* = 1’ for a U-turn, then we can get the corresponding formula as follows:

(20) $$a=\left\{ \begin{array}{lll} 0 & & \begin{array}{c} \left[70^{{}^{\circ}}\leq \theta \leq 120^{{}^{\circ}}\right] \end{array}\\ 1 & & \begin{array}{c} \left[160^{{}^{\circ}}\leq \theta \leq 210^{{}^{\circ}}\right] \end{array} \end{array}\right.$$

#### 3.5.4. Determine the Dangerous Steering Maneuvers Based on Weather Conditions

**Figure 22:**
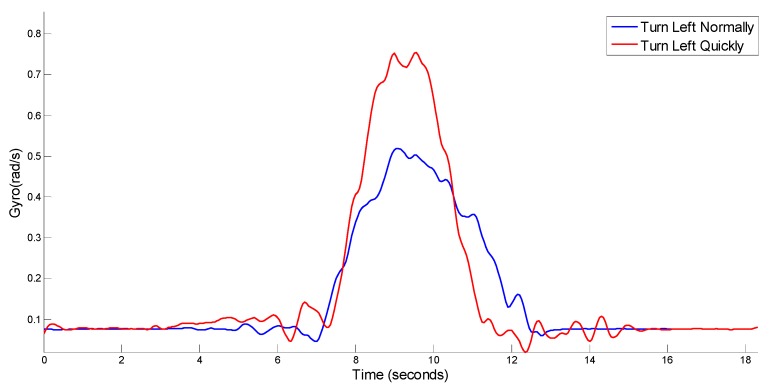
Gyro amplitude of different steering maneuvers.

The difference between a dangerous steering maneuver and a normal steering maneuver lies in the peak of the vehicle’s angle. As shown in Figure 22, it can be clearly seen that the value of vehicle’s angular velocity of a sudden left-turn is much larger than that of a normal left-turn. Therefore, after determining the situation of turns, lanes-changes and U-turns by matching the vehicle’s angular velocity and the steering angle of vehicle’s heading, we can move on to recognizing dangerous vehicle maneuvers by the method of setting the vehicle’s angular velocity thresholds. However, when setting of this threshold one needs to consider many factors, including the impact of road conditions, as a particularly important element. For example, the slippery roads or bad road visibility caused by rain, snow or fog and some other behaviors, including normal turns or lane-changes, may lead to traffic accidents. In those conditions, the definition of the threshold of angular velocity for dangerous vehicle maneuvers should be lower than for normal weather.

It is very important to determine the threshold of the vehicle’s angular velocity according to the different weather conditions. After collecting and averaging the data of the vehicle’s angular velocities of sudden turns, sudden lane-changes and sudden U-turns, and with numerous experiments, we define the thresholds of sudden turns, sudden lanes-changes and sudden U-turns in sunny days (under good road conditions) as 0.65, 0.45, and 0.75 rad/s, respectively. When the peak of the vehicle’s angular velocity is greater than the above thresholds, it is possible to further recognize the sudden turn, sudden lane-change and sudden U-turn maneuvers.

Similarly, we conducted the same experiment on rainy days, snowy days and foggy days, and then obtained the corresponding threshold by averaging the angular velocity. Based on the above data, we can preliminarily set the corresponding thresholds of a vehicle’s angular velocity under different weather conditions. The situation is summarized in Table 2.

### 3.6. Steering Maneuver Recognition Process

The steering maneuver recognition process can be described as follows: first, the occurrence of steering maneuvers is judged by the endpoint detection algorithm. Second, the turns, the lane-changes and the U-turns are determined by the matching algorithm of vehicle’s angular velocity and the steering angle of the vehicle. Meanwhile, whether the results belongs to the irresponsible steering maneuvers depends on the result of the turn light detection. Finally, we further recognize the conditions of sudden turns, rapid lane-changes and sudden U-turns according to the real-time weather, and the peak of the vehicle’s angular velocity, so as to output the recognition results. Examples of the necessary pseudo-code are as follows (Algorithm 1).

In the above recognition progress, the input parameters are the waveform’s short-term energy state state value, the vehicle’s angular velocity data Gyro, the short-term energy data $E_{n}$, the number of templates numsModule, the steering angle of vehicle’s heading Yaw, the threshold of vehicle’s angular velocity Gyro_t and the sign of turning on the turn light Turn_flag. Three states of the $E_{n}$ in the part of endpoint detection are shown in the 4th and 14th line, that is, the static section, the transition section and the driving sections mentioned in Section 3.5.1. Through the high $E_{high}$ and low thresholds $E_{low}$ and the interval of shortest time MinHoldTime, we can switch the three states above, and record the start and end points of the energy waveform. In the process of matching the vehicle’s angular velocity, the distance data distArray is defined as the 23rd line. The purpose is to store the matching results between the testing template to be measured and reference template of the vehicle’s angular velocity, that is, the cumulative distance of D(i,j) of the matching between the testing template to be measured and the reference template. The fast DTW method in the 26th line is the fast and dynamic time warping algorithm. The input parameters are the starting point and the ending point of the data of vehicle’s angular velocity. The minimum matching distance is output from the distArray in the 28th line. It is the reference corresponding with the reference template, which is mostly similar to the testing template to be measured, that is, it’s the matching result of the vehicle’s angular velocity. In the 29th line, we use the method of weather() to obtain the real-time weather data, so as to determine the angular velocity thresholds. According to the data in the 30th line, we can determine whether the turn signal is turned on, in accordance with the turn signal mark. According to the vehicle’s angular velocity threshold data in the 31st–34th lines, we can determine whether it is an instance of unscrupulous dangerous driving behavior, while the data of the 31st to 34th line are the thresholds of vehicle’s angular velocity, determining whether it is a dangerous vehicle maneuver, while the 34th–38th lines indicate that when the turn signal is turned on, we can determine whether the vehicle maneuver is a normal vehicle maneuver or a dangerous vehicle maneuver, through the vehicle’s angular velocity threshold.
**Algorithm 1.** Recognition Process Algorithm of Steering Maneuver1: **Begin:**2: Inputs: State, Gyro, En, numsModule, Yaw, Gyro_t, Turn_flag3: switch State4:  case Slience and Excess5:   if En > En_low6:    State < −Excess7:    Record the holdTime and startPoint8:    else if En < En_high9:    State < −Bump10:    Record the start time and holdTime11:    else12:    State < −Slience13:    clear the holdTime14:    case Bump15:    if En > En_low16:    Record the holdTime17:   else18:    if holdTime < MinHoldTime19:     State < −Slience20:     clear the holdTime21: endPoint = startPoint + holdTime22:23: distArray[numsModule]24: for(times = 1;times < numsModule;times++)25:  {26:   distArray[times] = fastDTW (Gyro.startPoint, Gyro.endPoint, ref_Gyro)27:  }28: driveResult = min(distArray) and Yaw29: Gyro_t = Weather()30: If Turn_flag == No31:  If Gyro > Gyro_t32:   driveResult = Sharp_CarelessDriving33:  else34:   driveResult = CarelessDriving35: else36:  If Gyro > Gyro_t37:   driveResult = SharpDriving38:  else39:   driveResult = NomalDriving


## 4. Experimental Results

### 4.1. Experimental Design

**Figure 23:**
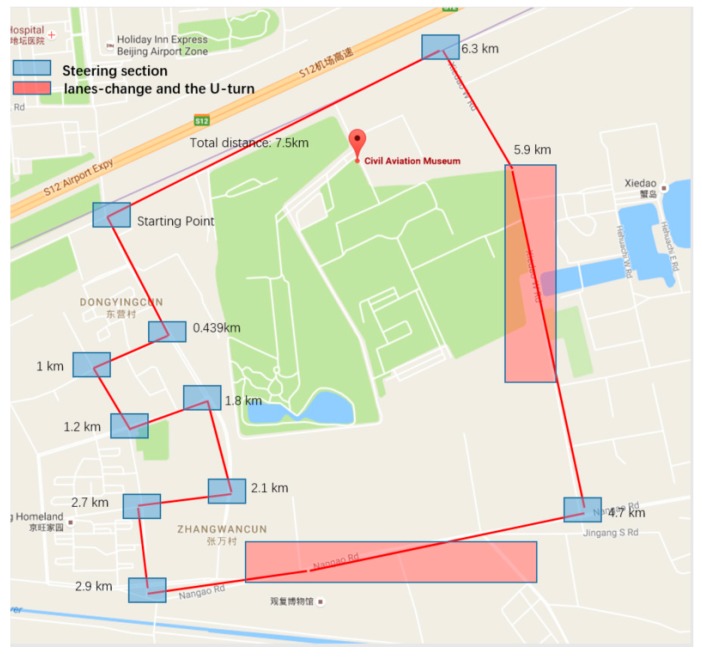
Pavement environment.

In this paper, the sensors of an iPhone6 were used for the collection of data from vehicle maneuvers. In order to verify the recognition accuracy of the recognition algorithm designed in this paper, we tested it by driving on the road. As shown in Figure 23, the blue part of the graph represents the road section used for testing the turn section, while the red part represents the one for testing the lane-changes and the U-turns.

Here are the calculation methods for the relevant indicators:

(21) $$TPR=\frac{TP}{P}=\frac{TP}{\left(TP+FN\right)}$$

(22) $$FPR=\frac{FP}{N}=\frac{FP}{\left(FP+TN\right)}$$

True Positives (*TP*) denotes the number of features that are predicted to be positive samples and are actually positive samples, while False Positives (*FP*) denotes the number of features that are predicted to be positive samples and are actually negative samples, True Negatives (*TN*) denotes the number of features predicted to be negative that actually are negative samples, False Negatives (*FN*) denotes the number of features that are negative samples and are actually positive samples. True Positive Rate (*TPR*) denotes the probability correctly predicting an actual positive sample, False Positive Rate (*FPR*) denotes the probability that an actual negative sample is erroneously predicted to be a positive sample.

### 4.2. Reference Template for Steering Maneuvers

In this paper, the angular velocity matching algorithm needs to store the vehicle’s angular velocity in advance. The reference template of vehicle’s angular velocity waveforms given in Section 3.5.1 is highly characteristic and can directly reflect a certain steering maneuver. To guarantee the accuracy of the reference template, a single vehicle and a single driver were applied to the measurement in this paper. A reference angular velocity template is obtained in the testing environment described above.

**Figure 24:**
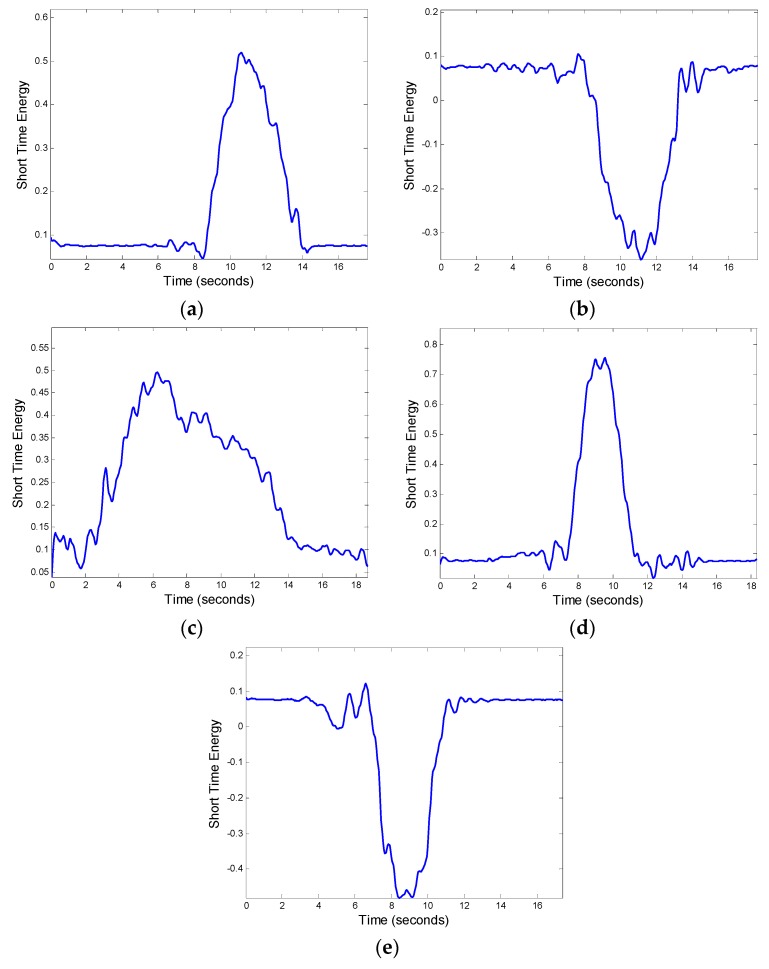
Steering pattern templates. (**a**) Turn left normally; (**b**) Turn right normally; (**c**) U-turn normally; (**d**) Left lane normally; (**e**) Right lane normally.

Five sets of reference templates were measured for each steering maneuver. The duration of each set of reference templates was judged by the endpoint detection mentioned in Section 3.5.1. After obtaining the average duration, the average angular velocity corresponding to various time points was calculated by the starting point and average duration of each group. Finally, we can obtain the average value of the reference template. Figure 24 shows the average values of the measured and calculated reference templates on a sunny day. These average values are for a normal left-turn, normal right-turn, normal left U-turn, and changing to the left lane and to the right lane, respectively.

After obtaining the vehicle maneuvers reference template, in the online phase, we can use the matching algorithm FastDTW in Section 3.5.2 to judge if a vehicle maneuver is a left turn, right turn, turn around, left lane or right lane change. The threshold set in Section 3.5.4 can be judged to be a sharp turn, a sudden U-turn, emergency road maneuver or other driving behavior.

### 4.3. Analysis and Comparison of Experimental Results

**Figure 25:**
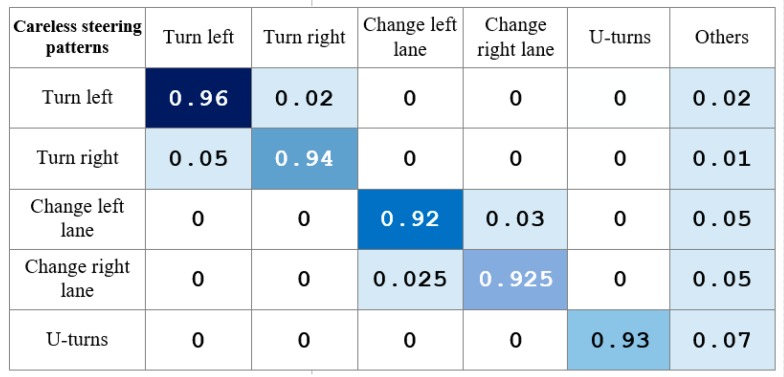
Steering recognition results.

As shown in Figure 25, our system has a pretty high accuracy rate in the recognition of dangerous steering maneuvers. Specifically, the accuracy rate of our system reaches 96% in recognizing dangerous left-turns, and 94% in recognizing dangerous right-turns. 

In the recognition of dangerous lane-changes to the left, the accuracy rate of our system reaches 92%; while for recognizing dangerous lane-changes to the right, the accuracy rate reaches 92.5%. Finally the accuracy rate of our system reaches 93% in recognizing dangerous U-turns.

The angular velocity waveform characteristics are relatively strong when the vehicle changes lanes, and we can exactly obtain the matching consequences when matching the angular velocity. The angular velocity threshold value can be set effectively in combination with the real-time weather, so as to make the accuracy rate and false positive rate of the steering recognition reach reasonable levels. In the U-turn recognition, we have introduced the vehicle’s heading turning angle, bringing about a high rate of U-turn recognition. 

### 4.4. The Recognition Accuracy of Different Smartphone Positions

**Figure 26:**
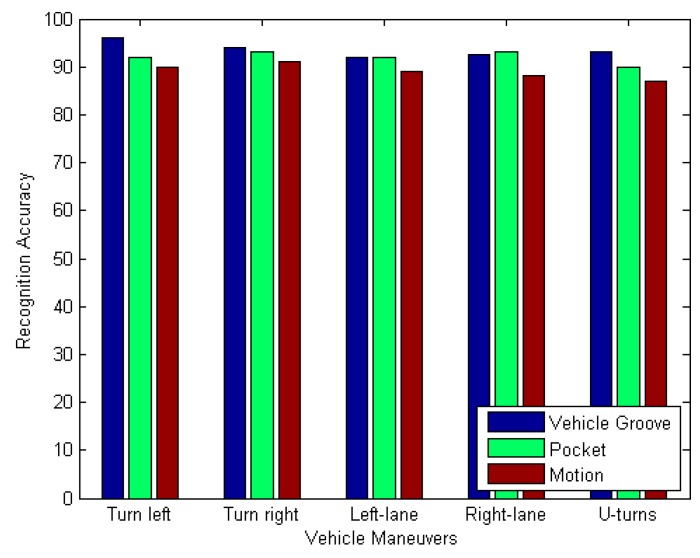
Steering recognition results.

According to Section 3.3.3, we know that when the mobile phone in the car is in an uncertain attitude, the gyro axis recorded data is often distributed in the three axis of the sub-vector data, which cannot visually reflect the vehicle angular velocity changes, so we use the coordinate system alignment algorithm to obtain the vehicle’s true angular velocity. We now validate the validity of the coordinate alignment algorithm by obtaining the recognition accuracy of the driving behavior for different positions of the mobile phone. We put the phone on the car in the dashboard slot, the driver’s pocket, as well as during the process of changing the location of the mobile phone to simulate the mobility of mobile phones. As shown in Figure 26, although the position of the mobile phone is different, the accuracy of the obtained vehicle maneuvers is almost the same, which proves the validity of our coordinate alignment algorithm.

### 4.5. The Recognition Accuracy of Different Vehicles and Drivers

To ensure the adaptability of the system, we first obtained the average reference template for the two Nissan Novelty and Honda Si Core Rui vehicle types, and recognized the vehicle maneuvers on the two vehicles. Finally, we used the reference template to recognize vehicle maneuvers on a Polo GTI vehicle type, and in order to take account of the differences of the driver’s operating habits, we use three volunteers to drive in the Honda Si Core Rui car.

**Figure 27:**
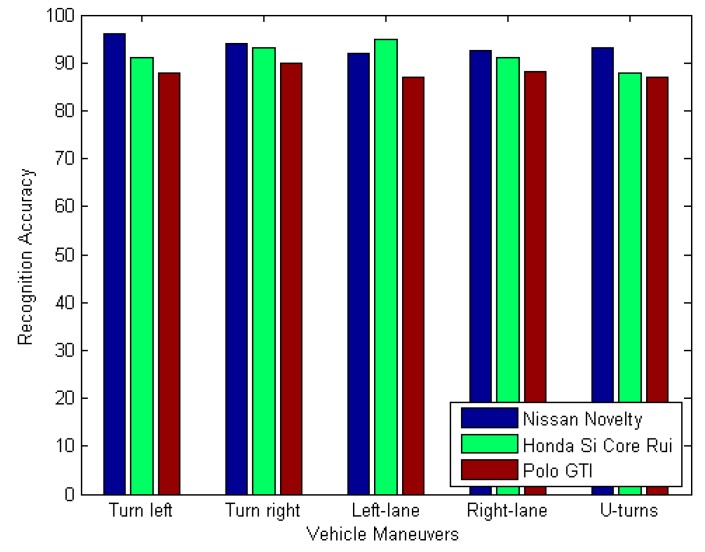
Different vehicle recognition results.

As shown in Figure 27, despite the fact reference templates for other types of vehicles were used by the Polo GTI, the exact average rate of identification is 90%, indicating that our system is adaptive.

### 4.6. Comparison with Other Systems

**Figure 28:**
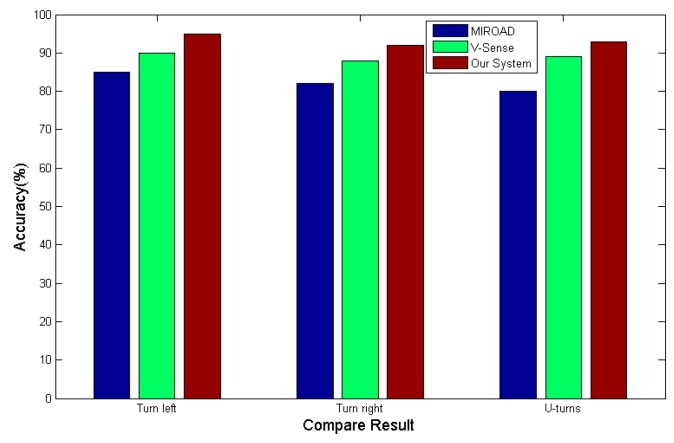
Steering maneuver comparison results.

We compare our system with the compass-based MIROAD to calculate the steering angle of vehicles and the V-Sense based on a mining algorithm of the vehicle’s angular velocity in the time domain. As shown in Figure 28, the compass is easily affected by the environment, resulting in measurement errors, therefore, the accuracy rate of the algorithm in this paper is higher than that of using the compass in the recognition of U-turns, while in the recognition of turning around and changing lanes, V-Sense uses the mining algorithm of the vehicle’s angular velocity in the time domain, but due to the differences in the mastery and proficiency of each driver, the waveforms of vehicle angular velocity will be different, and as shown in the figure, the accuracy rate of our system is slightly higher than that of the V-Sense method. Therefore, we can conclude that our algorithm for vehicle maneuvers has higher performance than the other algorithms in the same period.

## 5. Conclusions

In this paper, we mainly study recognition system for dangerous vehicle maneuvers based on smartphone sensors. As the location of the smartphone is not fixed in the vehicle, the data of vehicle’s steering angular velocity, which is collected by the smartphone’s inertial sensor, can’t directly reflect the driving behaviors of the vehicles. Therefore, we design an alignment method for the coordinate systems of the vehicle and the smartphone. Some of the methods recognizing steering maneuvers are based on smartphones, but they only use matching of a single factor, like the vehicle’s angular velocity, or only jointly make use of multi-sensors for the recognition, but these methods for the recognition for driving behaviors are not comprehensive enough. On the basis of these facts, a novel recognition algorithm for steering maneuvers is proposed in this paper. The steering maneuvers can be recognized by combining the matching of angular velocity, steering angle of the vehicle’s heading, weather factors and the detection of vehicle’s turn signal use together. This paper designs a recognition system for dangerous steering maneuvers with smartphones and it proves that the recognition strategy for steering maneuvers designed in this paper has good accuracy and low false alarm rate.

## Figures and Tables

**Table 1:** Classification of Driving Behaviors.

No.	Names	Description
1	Normal turns	Normal maneuvers of turns
2	Normal lane-changes	Normal maneuvers of changing lanes
3	Normal U-turns	Normal maneuvers of U-turns
4	Sudden turns	Sudden turns with turn signal on
5	Sudden lane-changes	Sudden lane changes with turn signal on
6	Sudden U-turns	Sudden U-turns with turn signal on
7	Turns without scruple	Normal turns or Sudden turns with turn signal off
8	Lane-changes without scruple	Normal lane-changes or Sudden lane-changes with turn signal off
9	U-turns without scruple	Normal U-turns or Sudden U-turns with turn signal off

**Table 2:** Dangerous driving behavior angular velocity thresholds.

Driving Behaviors	Sunny Days	Rainy Days	Sonwy Days	Foggy Days
Sudden Turns	0.65 rad/s	0.5 rad/s	0.35 rad/s	0.45 rad/s
Sudden lane changes	0.45 rad/s	0.35 rad/s	0.15 rad/s	0.3 rad/s
Sudden U-turns	0.75 rad/s	0.4 rad/s	0.2 rad/s	0.35 rad/s

## References

[1] Tefft B.C. (2010). Car Crashes Rank Among the Leading Causes of Death in the United States–Impact Speed and a Pedestrian’s Risk of Severe Injury or Death.

[2] Jain A., Koppula H.S., Raghavan B., Soh S., Saxena A. Car that knows before you do: Anticipating maneuvers via learning temporal driving models. Proceedings of the IEEE International Conference on Computer Vision.

[3] Jiang Y., Qiu H., McCartney M., Halfond W.G.J., Bai F., Grimm D., Govindan R. Carlog: A platform for flexible and efficient automotive sensing. Proceedings of the 12th ACM Conference on Embedded Network Sensor Systems.

[4] Jiang Y., Qiu H., McCartney M., Sukhatme G., Gruteser M., Bai F., Grimm D., Govindan R. CARLOC: Precisely Tracking Automobile Position. Proceedings of the 13th ACM Conference on Embedded Networked Sensor Systems.

[5] Mitrović D. (2005). Reliable method for driving events recognition. IEEE Trans. Intell. Transp. Syst..

[6] Hanggoro A., Putra M.A., Reynaldo R., Sari R.F. Green house monitoring and controlling using Android mobile application. Proceedings of the 2013 International Conference on QiR (Quality in Research).

[7] Miranda-Moreno L.F., Chung C., Amyot D., Chapon H. A System for Collecting and Mapping Traffic Congestion in a Network Using GPS Smartphones from Regular Drivers. Proceedings of the Transportation Research Board 94th Annual Meeting.

[8] Incel O.D., Kose M., Ersoy C. (2013). A Review and Taxonomy of Activity Recognition on Mobile Phones. BioNanoScience.

[9] Tang F., You I., Tang C., Guo M. (2013). An efficient classification approach for large-scale mobile ubiquitous computing. Inform. Sci..

[10] Rodrigues J.J.P.C., Lopes I.M.C., Silva B.M.C., de La Torre I. (2013). A new mobile ubiquitous computing application to control obesity: SapoFit. Inform. Health Soc. Care.

[11] Wang Y., Yang J., Liu H., Chen Y., Gruteser M., Martin R.P. Sensing vehicle dynamics for determining driver phone use. Proceedings of the 11th Annual International Conference on Mobile Systems, Applications, and Services.

[12] Yang J., Sidhom S., Chandrasekaran G., Vu T., Liu H., Cecan N., Chen Y., Gruteser M., Martin R.P. Detecting driver phone use leveraging car speakers. Proceedings of the 17th Annual International Conference on Mobile Computing and Networking.

[13] Dai J., Teng J., Bai X., Shen Z., Xuan D. Mobile phone based drunk driving detection. Proceedings of the 4th International Conference on Pervasive Computing Technologies for Healthcare.

[14] White J., Thompson C., Turner H., Dougherty B., Schmidt D.C. (2011). WreckWatch: Automatic traffic accident detection and notification with smartphones. Mob. Netw. Appl..

[15] Johnson D.A., Trivedi M.M. Driving style recognition using a smartphone as a sensor platform. Proceedings of the 14th International IEEE Conference on Intelligent Transportation Systems (ITSC).

[16] Chen D., Cho K.T., Han S., Jin Z., Shin K.G. Invisible sensing of vehicle steering with smartphones. Proceedings of the 13th Annual International Conference on Mobile Systems, Applications, and Services.

[17] Eriksson J., Girod L., Hull B., Newton R., Madden S., Balakrishnan H. (2008). The Pothole Patrol: Using a Mobile Sensor Network for Road Surface Monitoring.

[18] Mednis A., Strazdins G., Zviedris R., Kanonirs G., Selavo L. (2011). Real Time Pothole Detection Using Android Smartphones with Accelerometers.

[19] Mohan P., Padmanabhan V., Ramjee R. (2008). Nericell: Rich Monitoring of Road and Traffic Conditions Using Mobile Smartphones.

[20] NHTSA. http://www.nhtsa.gov/.

[21] Zhou P., Li M., Shen G. Use it free: Instantly knowing your phone attitude. Proceedings of the 20th annual international conference on Mobile computing and networking.

[22] MatrixTransforms. https://developer.apple.com/library/content/documentation/AudioVideo/Conceptual/HTML-canvas-guide/MatrixTransforms/MatrixTransforms.html.

[23] Gubner J.A. (2006). Probability and Random Processes for Electrical and Computer Engineers.

[24] Lamel L.F., Rabiner L.R., Rosenberg A.E., Wilpon J. (1981). An improved endpoint detector for isolated word recognition. IEEE Trans. Acoust. Speech Signal Proc..

[25] Jalil M., Butt F.A., Malik A. Short-time energy, magnitude, zero crossing rate and autocorrelation measurement for discriminating voiced and unvoiced segments of speech signals. Proceedings of International Conference on Technological Advances in Electrical, Electronics and Computer Engineering.

[26] Keogh E., Ratanamahatana C.A. (2005). Exact indexing of dynamic time warping. Knowl. Inf. Syst..

[27] Berndt D.J., Clifford J. (1994). Using Dynamic Time Warping to Find Patterns in Time Series. KDD Workshop.

[28] Keogh E.J., Pazzani M.J. (2001). Derivative Dynamic Time Warping. SDM.

[29] Salvador S., Chan P. (2007). Toward accurate dynamic time warping in linear time and space. Intell. Data Anal..

[30] Kim J., Kim S., Schaumburg E., Sims C.A., Schaumburg E. (2008). Calculating and using second-order accurate solutions of discrete time dynamic equilibrium models. J. Econ. Dyn. Control.

[31] Ajtay D., Weilenmann M., Soltic P. (2005). Towards accurate instantaneous emission models. Atmos. Environ..

[32] Salvador S., Chan P. FastDTW: Toward Accurate Dynamic Time Warping in Linear Time Space. Presented at the 3rd Workshop on Mining Temporal and Sequential Data.

